# Follicle-stimulating hormone promotes energy metabolism in bovine granulosa cells through the FOXO1-ISG15 pathway to maintain the development of multiple follicles

**DOI:** 10.1186/s40104-026-01486-9

**Published:** 2026-08-12

**Authors:** Huan Wang, Zhihui Hu, Huabin Zhu, Chao Wang, Jing An, Shanjiang Zhao

**Affiliations:** 1 Key Laboratory of Animal Genetics, Breeding and Reproduction of Ministry of Agriculture and Rural Affairs, Institute of Animal Science, Chinese Academy of Agricultural Sciences, Beijing, 100193 China; 2 State Key Laboratory of Animal Biotech Breeding, College of Biological Sciences, China Agricultural University, Beijing, 100193 China; 3 College of Animal Science and Technology, Northwest Agriculture and Forest University, Yangling, Xianyang, 712100 China

**Keywords:** Energy metabolism, Follicular development, FOXO1, Granulosa cell proliferation, pFSH

## Abstract

**Background:**

As a central regulator of follicular development, follicle-stimulating hormone (FSH) is widely applied to induce synchronous multi-follicular growth in assisted reproductive technologies and livestock breeding. However, the molecular mechanism linking FSH signaling to synchronous multi-follicular development remains poorly defined. This study aimed to investigate the molecular mechanism by which porcine purified pituitary FSH (pFSH) promotes translation of multiple follicles.

**Results:**

Here, we established a mammalian model of multi-follicular development using pFSH and integrated single-cell transcriptomics with functional analyses, which uncovered a regulatory axis governing the metabolism and proliferation of granulosa cells (GCs). Treatment with pFSH significantly increased ovarian weight, the number of antral follicles—particularly small antral follicles (3–5 mm)—and circulating estradiol levels. Single-cell RNA sequencing of GCs from small antral follicles revealed that pFSH markedly enhanced the expression of genes involved in energy metabolism, especially the oxidative phosphorylation and mitochondrial electron transport chain pathways, while concurrently downregulating FOXO1 expression. Mechanistically, pFSH activated the PI3K/AKT pathway to drive phosphorylation of FOXO1 at Ser267, a modification that is required for GC proliferation and metabolic activation. Overexpression of the dephosphorylated mutant FOXO1(S267A) abolished the pFSH-induced increases in GC proliferation, glucose uptake, oxidative phosphorylation, and ATP production, highlighting a central role of FOXO1 phosphorylation in mediating FSH responses. RNA-seq and chromatin immunoprecipitation analyses further identified ISG15 as a direct transcriptional target of FOXO1. Upon binding to the promoter region, FOXO1 activates ISG15 expression, linking FSH signaling to an interferon-related pathway and metabolic regulation.

**Conclusions:**

Collectively, this study revealed the pivotal role of the pFSH–FOXO1–ISG15 regulatory axis in orchestrating GC metabolic reprogramming and synchronous multi-follicular development. These finding provide a conceptual framework to clarify how endocrine signals integrate with metabolic and immune pathways to regulate reproductive processes, with potential implications to improve assisted reproduction and livestock productivity.

**Supplementary Information:**

The online version contains supplementary material available at 10.1186/s40104-026-01486-9.

## Introduction

Follicular development is a highly synchronized process governed by endocrine signaling, with follicle-stimulating hormone (FSH) serving as a central regulator of follicular growth and selection. In monotocous species, such as humans and cattle, follicular development follows a wave-like pattern tightly controlled by dynamic fluctuations in circulating FSH levels. According to the “FSH threshold/window” theory [1], the onset of follicular growth is initiated when FSH concentrations exceed a certain minimum threshold during the early follicular phase, which recruits a cohort of antral follicles. However, this “window” is temporally limited to ensure the development and ovulation of typically only one dominant follicle, whereas the others undergo atresia. In clinical and agricultural settings, exogenous FSH is administered to maintain supra-threshold concentrations to override this physiological constraint and enable synchronous multi-follicular development for human-assisted reproduction and superior livestock breeding [2, 3]. However, substantial inter-individual variability in response to FSH stimulation limits the efficiency and safety of assisted reproductive technologies [4]. Moreover, the molecular mechanisms underlying FSH-induced synchronous follicular development, particularly those contributing to differential responsiveness, remain poorly defined.

As essential mediators of follicular development, granulosa cells (GCs), along with hormonal cues, promote follicular growth and oocyte competence [5–7]. Beyond the classical roles in hormone synthesis and paracrine signaling, GCs provide essential metabolic support to the oocyte through gap junction-mediated exchange of nutrients and signaling molecules [8]. Accumulating evidence indicates that the metabolic state of GCs, including mitochondrial function, oxidative phosphorylation, and glucose metabolism, is a key determinant of oocyte quality and follicular fate. Dysregulation of energy metabolism of GCs, such as glucose metabolism, mitochondrial respiration, and glycolysis, has been implicated in reproductive disorders, such as aging-associated infertility and polycystic ovary syndrome, which are both characterized by impaired follicular development and reduced oocyte competence [8–10]. Although FSH is known to regulate multiple functions of GCs, whether and how FSH promotes multi-follicular growth through metabolic reprograming of GCs remains largely unknown.

FOXO1 (Forkhead box protein O1), the earliest identified member of the Forkhead box O family of transcription factors, plays a critical regulatory role in diverse cellular processes, including proliferation, differentiation, stress responses, and energy metabolism [11–14]. FOXO1 activity is tightly controlled by post-transcriptional modifications, particularly phosphorylation mediated by the PI3K/AKT pathway, which promotes nuclear exclusion and functional inactivation. Phosphorylated FOXO1 is translocated from the nucleus to the cytoplasm, where it is subsequently degraded and loses transcriptional activity [15, 16]. The regulatory role of FOXO1 in follicular development has garnered increasing attention in the field of reproductive biology [17, 18]. In porcine GCs, upregulation of FOXO1 under hypoxic conditions induces cell cycle arrest at the G0/G1 phase, which inhibits cellular proliferation [19]. In human GCs, FOXO1 upregulation under the pathological condition of polycystic ovary syndrome triggers mitochondrial dysfunction and apoptosis [20]. Notably, a recent study of ovine GCs revealed that FSH can modulate FOXO1 activity through Akt-dependent phosphorylation, thereby influencing glucose metabolism and cellular function [21]. However, these findings suggest that phosphorylated FOXO1 may also play a significant role in linking FSH signaling to metabolic regulation of GCs, thereby limiting synchronous multi-follicular development.

In this study, a mammalian model of multi-follicular development was established using pFSH and integrated with single-cell transcriptomics with functional analyses to elucidate the underlying regulatory mechanisms. We identified a previously unrecognized pFSH–FOXO1–ISG15 signaling axis that drives energy metabolism of GCs and supports synchronous multi-follicular development. These findings provide a conceptual framework to clarify how endocrine signals are coupled to metabolic and immune pathways during folliculogenesis, with potential implications for improving assisted reproduction and livestock productivity.

## Materials and methods

### Animals and the pFSH-induced multi-follicular development model

Six Holstein cows with similar age and body weight, free from reproductive disorders and other diseases affecting reproductive performance, were selected from the same population within a single dairy farm. The animals were randomly divided into an experimental group and a control group, with three cows per group. Controlled Internal Drug Release (CIDR) devices were inserted for estrus synchronization on d 0. Starting on d 5, cows in the experimental group received intramuscular injections of Porcine Pituitary Follitropin Extract (pFSH, Folltropin-V^®^, Canada) following a conventional superovulation protocol for two consecutive days, for a total of four injections, with volumes of 3 mL, 3 mL, 2 mL, and 2 mL, respectively. The control group received equivalent volumes of physiological saline. Throughout the treatment period, daily ultrasound examinations were performed on all cows to monitor follicular development. After the final injection, the cows were slaughtered, and ovarian tissues were collected.

Follicles of various diameters were rapidly collected from the ovaries and counted by mechanical disruption. Studies have shown that in cattle, FSH primarily acts on small antral follicles with a diameter of 3–5 mm; follicles within this size range are most sensitive to FSH fluctuations, and their growth is directly dependent on FSH stimulation [22]. Based on morphological criteria, small antral follicles with a diameter of 4 ± 0.2 mm that appeared developmentally active were selected for single-cell sequencing. Follicle selection criteria included: structurally intact follicles with abundant surface vasculature, clear follicular fluid, and an intact granulosa cell layer without significant shedding. To obtain a tissue suspension, follicles were minced, transferred together with follicular fluid into centrifuge tubes, and digested in digestion solution in a 37 °C water bath for 15 min. The digestion solution consisted of RPMI 1640 (Gibco, #11835030), Dispase (STEMCELL Technologies, #07923), Collagenase Type IV (NoninBio, NBS1004), and DNase I (Invitrogen, #18068015). Following digestion, a 40 μm cell strainer was used to obtain a single-cell suspension. Subsequently, the single-cell suspension was used to generate a barcoded single-cell RNA-seq library with the 10X Genomics Chromium Next GEM Single Cell 3' Reagent Kits v3.1. Sequencing was performed on the Illumina Novaseq high-throughput platform in PE150 paired-end mode. For each single-cell sequencing, samples from 3 individual cows (six follicles per cow) were sequenced separately, with no pooling of follicles across cows.

### Quality control

Raw sequencing data were processed using CellRanger (v7.1.0) software for reference genome alignment, data filtering, and unique molecular identifier (UMI) counting. The bovine (*Bos taurus*) genome (GCF_002263795.3, https://www.ncbi.nlm.nih.gov/datasets/genome/GCF_002263795.3/) was used as the reference genome for alignment. Subsequent quality control and filtering of the expression matrix were performed using Seurat (4.1.1) with the following criteria: the number of genes expressed per cell was between 200 and 6,000, and the percentage of mitochondrial gene expression was below 15%. Doublets were filtered out using DoubleFinder (v2.0.3).

### Dimensionality reduction and cell clustering

After quality control and doublet removal, single-cell transcriptomic data were processed using Seurat (v4.1.1). Raw UMI counts were normalized via the LogNormalize method with a scaling factor of 10,000. The top 2,000 highly variable genes were identified using variance stabilizing transformation, followed by data scaling with regression of mitochondrial gene content and total UMI counts to mitigate technical confounders. Principal component analysis was performed, and the top 30 statistically significant principal components were selected via JackStraw permutation test (adjusted *P* < 0.05) and elbow plot for downstream analyses. Unsupervised cell clustering was performed using the Louvain algorithm with optimized resolution (0.4–1.2), with results visualized by Uniform Manifold Approximation and Projection (UMAP) and t-Distributed Stochastic Neighbor Embedding (t-SNE) using the selected 30 principal components. Cell types were annotated using canonical bovine ovarian marker genes, with cluster-specific markers validated via the Wilcoxon rank-sum test. Annotated granulosa cells were extracted for independent subclustering, and differential expression analysis between control and experimental groups was performed for each granulosa subcluster using the Wilcoxon rank-sum test. Functional enrichment analysis of significant differentially expressed genes (|log_2_(FC)| ≥ 0.58, Benjamini–Hochberg adjusted *P* < 0.05) was conducted using clusterProfiler with matched Bos taurus ARS-UCD1.3 genome annotation.

### Cell culture

The primary granulosa cells used in this study were isolated and established from ovaries of Holstein cows obtained from a slaughterhouse. Follicular fluid containing granulosa cells was aspirated from well-developed small antral follicles (3–5 mm in diameter) using a syringe. After removing oocytes from the follicular fluid using a 40 μm cell strainer, primary granulosa cell lines were established. All cells were cultured in DMEM/F12 medium supplemented with 10% fetal bovine serum (FBS) and 1% penicillin–streptomycin (PS) at 37 °C in a 5% CO₂ atmosphere. To validate the regulatory effect of pFSH on granulosa cells in vitro, cells were treated with 0.07 IU/mL pFSH (Folltropin-V^®^, Canada) for 12 h. In this experiment, the mutant oeFOXO1 and FOXO1(S267A) were transfected into granulocytes using lipid-mediated transfection (Invitrogen, L3000015). Prior to subsequent functional experiments, the optimal dose of the FOXO1 overexpression plasmid was determined. Granulosa cells were transfected with 0.5, 1.0, or 2.5 μg plasmid, and FOXO1 expression at both the mRNA and protein levels was assessed 12 h post-transfection. Based on overexpression efficiency and cell morphology (Fig. S1), 1.0 μg plasmid was selected for all subsequent experiments.

### Immunofluorescence

A total of 1 × 10^6^ cells were seeded onto coverslips placed in 6-well plates and cultured for 6 h. Following FSH treatment for 12 h, cells were fixed with 4% paraformaldehyde for 30 min at room temperature. Permeabilization was performed using PBS containing 0.3% Triton X-100 for 15 min. After three washes with PBS, cells were blocked with PBS containing 5% bovine serum albumin for 60 min at room temperature. FOXO1 primary antibody (Cell Signaling Technology, #2880, 1:100 dilution) was incubated overnight at 4 °C, followed by secondary antibody incubation for 2 h at room temperature. Coverslips were mounted using ProLong™ Diamond Antifade Mountant with DAPI (Invitrogen, P36961) and observed under a confocal microscope (Nikon Eclipse80i). Immunofluorescence staining intensity was quantified using Image J analysis.

### Western blotting and antibodies

Cellular proteins were extracted using Invent® RIPA lysis buffer (Invent, IN-WB001) supplemented with 0.1% SDS, protease inhibitors, and phosphatase inhibitors. After three repeated freeze–thaw cycles, the lysates were incubated at 100 °C for 10 min to denature the proteins. Proteins were separated by SDS-PAGE and transferred onto nitrocellulose membranes (Bio-Rad, #162-0115). Membranes were incubated with the corresponding primary antibodies overnight at 4 °C, followed by incubation with the appropriate secondary antibodies for 2 h at room temperature. Protein bands were visualized using an ECL detection kit (Bio-Rad, #170-5060) and chemiluminescence was detected with the ChemiDoc MP Imaging System (Bio-Rad). Band intensities were quantified by densitometry analysis using Image J software. The antibodies used in this experiment were as follows: FOXO1 (Cell Signaling Technology, #2880, 1:1,000); Phospho-FOXO1(Ser256) (Cell Signaling Technology, #2880, 1:1,000); AKT (Cell Signaling Technology, #9272, 1:1,000); Phospho-AKT (Ser473) (Cell Signaling Technology, #9271, 1:1,000); PI3K (Cell Signaling Technology, #4292, 1:1,000); OXPHOS (Abcam, ab110413, 1:1,000); β-Actin (Proteintech, #81,115-1-RR, 1:2000); Anti-Rabbit IgG, HRP-linked Antibody (Cell Signaling Technology, #7074, 1:1,000).

### IncuCyte and EdU cell proliferation assays

A total of 1 × 10^6^ cells were seeded into each well of 12-well plates and cultured in 2 mL of DMEM/F12 medium supplemented with 10% FBS and 1% PS. Immediately after pFSH treatment or overexpression, the 12-well plates were placed into an IncuCyte^®^ live-cell analysis system (Incucyte SX5, Sartorius). Images were captured at 3 h intervals. After 24 h of observation, cell confluence was obtained and used to generate cell proliferation curves.

For EdU incorporation assays, a total of 1 × 10^6^ cells were seeded into each well of 6-well plates and cultured in 1 mL of DMEM/F12 medium containing 10% FBS and 1% PS. After pFSH treatment or overexpression for 12 h, EdU incorporation was detected according to the manufacturer's instructions for the BeyoClick™ EdU-488 Cell Proliferation Assay Kit (Beyotime, C0071L). An equal volume of 5-ethynyl-2'-deoxyuridine (EdU) was added to each well and incubated for 2 h. Cells were then fixed with 1 mL of 4% paraformaldehyde (Beyotime, P0099) for 15 min at room temperature. After three washes with PBS, cells were incubated with 1 mL of PBS containing 0.3% Triton X-100 for 15 min at room temperature. Following one wash with PBS, 0.5 mL of Click reaction cocktail was added and incubated for 30 min at room temperature protected from light. After three washes with PBS, nuclei were stained with Hoechst 33342 (Sigma, B2261) for 10 min at room temperature protected from light. Results were observed using an inverted fluorescence microscope, and counting analysis was performed using Image J software.

### qRT-PCR

Total RNA was extracted from cells using the FastPure Cell/Tissue Total RNA Isolation Kit (Vazyme, RC101-01). The complementary DNA was synthesized using a QuantStudio5 real-time fluorescent quantitative PCR system (Applied Biosystems, Waltham, MA, USA) in a total volume of 15 µL. The reaction system consisted of 10 µL 2 × SYBR green PCR buffer (ABI, 4368708, Raleigh, USA), 1 µL of 10 µmol/L PCR forward primer, 1 µL of 10 µmol/L PCR reverse primer, 1 µL of template, and 6 µL of DNase/RNase-free water. The reaction procedure was one cycle, denaturation at 50 °C for 2 min, denaturation at 95 °C for 2 min, followed by 40 cycles of amplification, denaturation at 95 °C for 15 s, and annealing extension at 60 °C for 1 min, followed by melt curve analysis in triplicate. The expression data were normalized to β-actin using the 2^−∆∆CT^ method. The sequences of the primers used are shown in Table S1.

### Mitochondrial stress test

A total of 4 × 10^4^ cells were seeded into XFe96 FluxPaks (Agilent, #102416) and cultured in DMEM/F12 medium containing 10% FBS and 1% PS for 6 h. Following this, cells were subjected to pFSH treatment or overexpression for 12 h, ensuring a final volume of 80 μL per well. Subsequently, the medium was replaced with XF Base Medium supplemented with 10 mmol/L glucose (Sigma, G7528), 2 mmol/L L-glutamine (Sigma, G8540), and 1 mmol/L sodium pyruvate (Sigma, S8636). Cells were incubated in a non-CO₂ incubator (Agilent, XF Prep) for 1 h at 37 °C. The mitochondrial oxygen consumption rate (OCR) was measured using a Seahorse XFe96 Analyzer (Agilent) under basal conditions and following sequential injections of 3 μmol/L oligomycin (ATP synthase inhibitor; Abcam, ab141829), 1 μmol/L carbonyl cyanide-p-trifluoromethoxy phenylhydrazone (FCCP; mitochondrial uncoupler; Sigma, C2920), and a mixture of 1.5 μmol/L antimycin A (respiratory chain inhibitor; Abcam, ab141904) and 3 μmol/L rotenone (Sigma, R8875). Cell counting for data normalization was performed using the Cell Normalization Fluorescent Assay Kit (Alicelligent, ALN23013). Mitochondrial respiration parameters were calculated according to the standard Seahorse XF Cell Mito Stress Test workflow. Basal respiration was defined as the baseline OCR prior to oligomycin injection after correction for non-mitochondrial respiration. ATP-linked respiration was calculated as the difference between basal OCR and OCR measured after oligomycin treatment: ATP-linked respiration = Basal OCR − Oligomycin OCR. Maximal respiration was calculated as the OCR following FCCP injection minus non-mitochondrial respiration. Spare respiratory capacity was determined as maximal respiration minus basal respiration.

### Glucose uptake assay

Glucose uptake levels were measured using a glucose uptake fluorescence assay kit (Beyotime, S0561S). A total of 1 × 10^6^ cells were seeded into each well of 6-well plates and cultured in 2 mL of DMEM/F12 medium containing 10% FBS and 1% PS. After pFSH treatment or overexpression for 12 h, 20 μL of Glucose Uptake Inhibitor was added to each well, and cells were incubated for 30 min at 37 °C in a 5% CO₂ atmosphere. Subsequently, 1 mL of 2-deoxy-2-{(7-nitro-2,1,3-benzoxadiazol-4-yl)amino}-D-glucose (2-NBDG) glucose uptake working solution was added to each well and incubated for 10 min at room temperature. Nuclei were then stained with Hoechst 33342 (B2261, Sigma) for 10 min at room temperature protected from light. Results were observed using an inverted fluorescence microscope, and counting analysis was performed using Image J software.

### ATP quantification

ATP levels in cells were measured using an Enhanced ATP Assay Kit (Beyotime, S0027). A total of 1 × 10^6^ cells were seeded into each well of 6-well plates. After pFSH treatment or overexpression for 12 h, cells were lysed by adding 200 μL of lysis buffer per well according to the manufacturer's instructions. Subsequently, 100 μL of ATP detection working solution was added to each well of a 96-well plate (Corning, 3922) and incubated for 10 min at room temperature. After adding 20 μL of sample, the luminescence was measured using a multimode microplate reader (Tecan, Infinite 200 Pro). Protein concentration in each well was determined using an Enhanced BCA Protein Assay Kit (Beyotime, C2003S). ATP concentrations in the samples were calculated based on a standard curve and then normalized to protein concentration. ATP levels were expressed as nmol/mg protein.

### Transcription factor prediction and chromatin immunoprecipitation (ChIP)

Transcription factors for ISG15 were predicted using JASPAR (https://jaspar.elixir.no/) with a relative profile score threshold set to 90%. The predicted binding sites were validated using the SimpleChIP^®^ Plus Enzymatic Chromatin IP Kit (Magnetic Beads) (CST, #9005). Granulosa cells, treated with pFSH or subjected to overexpression for 12 h, were incubated with 1% formaldehyde (Macklin, F809702) for 10 min at room temperature to cross-link proteins to DNA. The cross-linking reaction was quenched by adding Glycine Solution (10X) (CST, #7005) and incubating for 5 min at room temperature. Cells were lysed according to the kit manufacturer's instructions, and chromatin was digested with Micrococcal Nuclease (CST #10011) for 20 min at 37 °C. The samples were then subjected to three rounds of sonication (SCIENIZ, JY92-2) at 400 W power, with 25 s pulses and 50 s intervals, to shear chromatin to fragments of 150–900 bp. For immunoprecipitation, samples were incubated overnight at 4 °C with rotation with antibodies suitable for ChIP: the positive control group received 10 µL of Histone H3 (D2B12) XP^®^ Rabbit mAb (ChIP Formulated) (CST, #4620), the negative control group received 10 µL of Normal Rabbit IgG (CST, #2729), and the experimental group received 10 µL of FOXO1 (C29H4) Rabbit Monoclonal Antibody (CST, #2880). ChIP-Grade Protein G Magnetic Beads (CST, #9006) were added, and samples were incubated for 2 h at 4 °C with rotation for immunoprecipitation. Complexes were eluted by adding ChIP elution buffer and incubating for 30 min at 65 °C. Protein-DNA cross-links were reversed by incubation with Proteinase K overnight at 65 °C. DNA was purified using the spin columns provided in the kit. The purified DNA was used for PCR and RT-qPCR. The sequences of the primers used are shown in Table S1.

### Statistical analysis

We used SAS software version 9.2 (SAS, Cary, NC, USA) for statistical analysis. Differences between two groups were analyzed using a two-tailed Student's *t*-test. *P* < 0.05 was considered statistically significant. Results are shown as means ± standard deviation (SD). All experiments were repeated at least in three independent experiments.

## Results

### pFSH promoted ovarian synchronous multi-follicular development in cattle

**Fig. 1:**
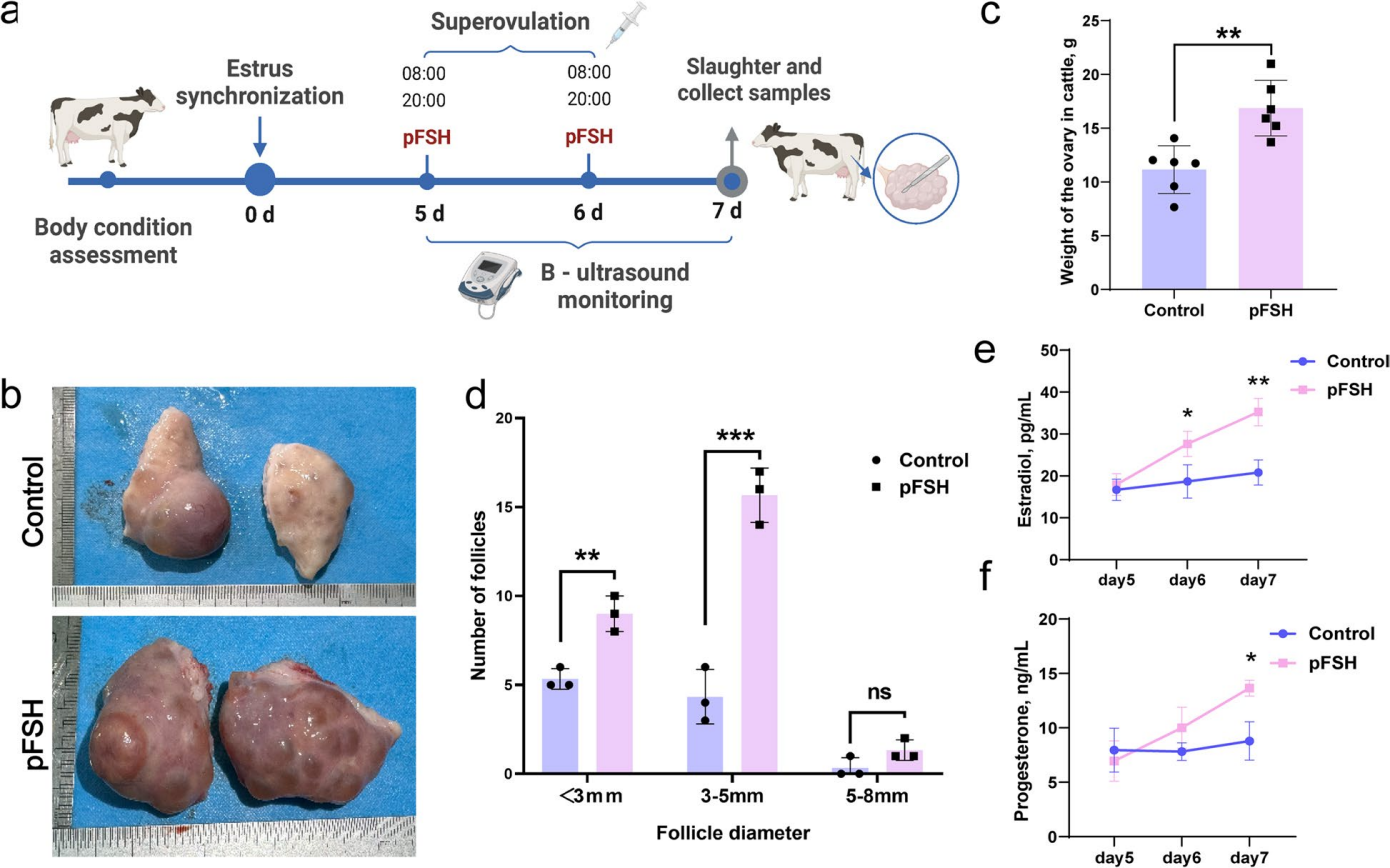
pFSH promotes the development of an increased number of small antral follicles. **a** Schematic diagram of the experimental treatment protocol. CIDR devices were inserted for estrus synchronization. After 5 d, cows in the experimental group received pFSH injections for 2 consecutive days in accordance with a conventional superovulation protocol, while the control group received equivalent volumes of physiological saline. **b** Overview of ovaries from the control and experimental groups. **c** Bar graph showing ovarian weight in the control and experimental groups. **d** Bar graph showing follicle counts in the control and experimental groups. **e** Line graph showing estradiol levels in the control and experimental groups. **f** Line graph showing progesterone levels in the control and experimental groups. ^*^P < 0.05; ^**^P < 0.01; ^***^P < 0.001; ns, non-significant

A bovine model of synchronous multi-follicular development was established to investigate the effect of pFSH. As compared to the control group, pFSH significantly increased ovarian weight (Fig. 1b and c) and particularly the number of small antral follicles (3–5 mm) (Fig. 1a and b), whereas serum estradiol levels were markedly elevated at 1 day after pFSH injection. At 2 days after pFSH injection, serum estradiol levels remained elevated in the experimental group, while progesterone levels were also significantly increased (Fig. 1e and f). Ultrasound monitoring further confirmed that small antral follicles (3–5 mm) were highly responsive to pFSH injection (Fig. S2).

### Single-cell transcriptomics revealed metabolic reprogramming of GCs

**Fig. 2:**
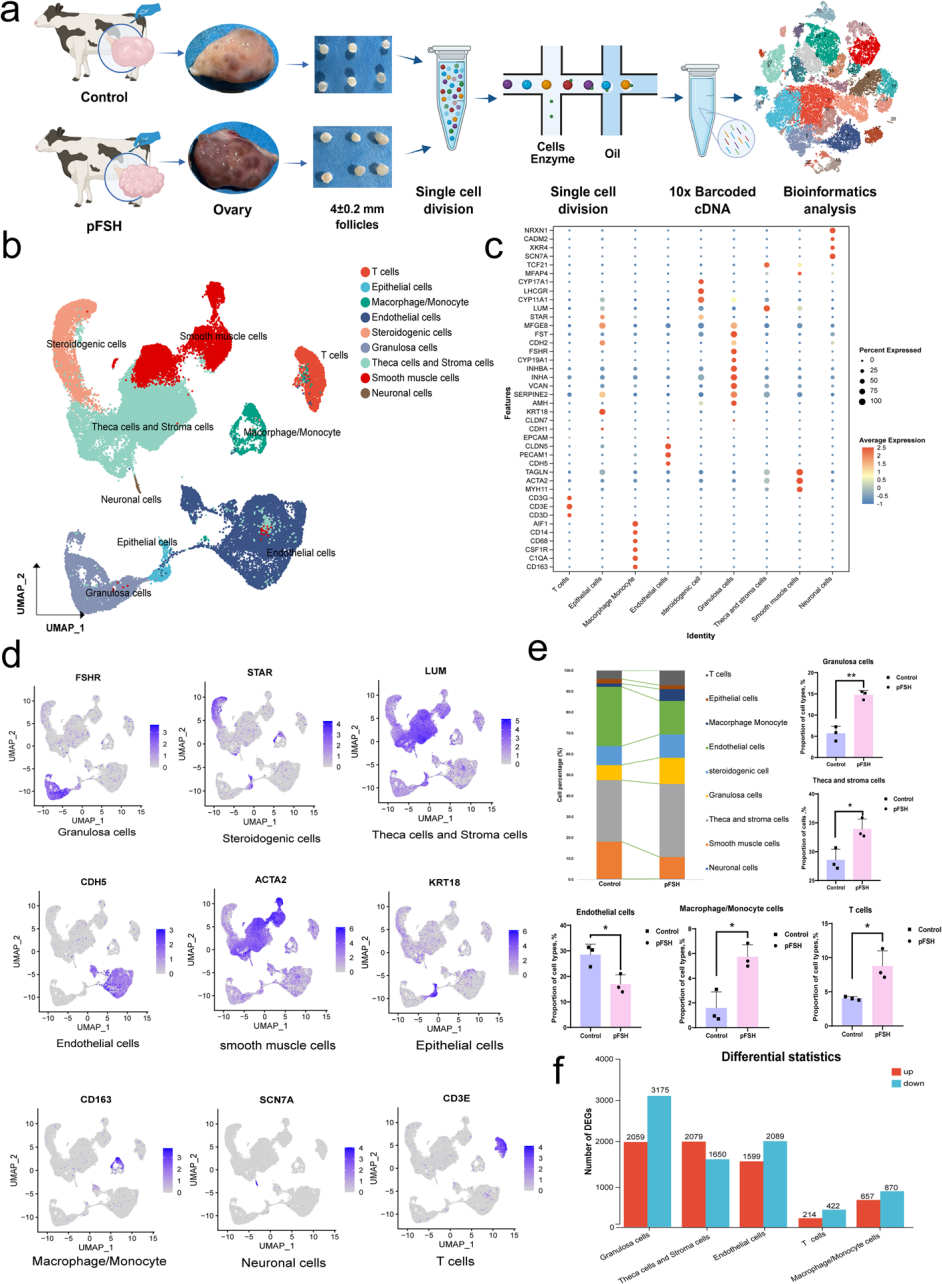
Transcriptome profiling of small antral follicles. **a** Schematic workflow of single-cell sequencing of small antral follicles. **b** UMAP plot showing specific clustering of major cell types from small antral follicles. Each point corresponding to a single cell is color-coded according to its cell type membership. **c** Bubble plot of differential expression analysis of the major cell types. **d** Feature plots demonstrating expression specificity of the signature genes across the small antral follicles cells. The expression level of each gene from none to high is indicated by a color gradient from light gray to dark blue. Blue dashed lines give boundaries of the main clusters of interest. **e** Cell proportions of each cell type in the control group and the pFSH-treated group, accompanied by statistical bar plots for the 5 cell types showing statistically significant differences in cell proportions between the two groups. **f** Statistical bar plots of DEGs between the control group and the pFSH-treated group in the 5 cell types with significant proportion alterations. Red bars represent significantly upregulated genes, and blue bars represent significantly downregulated genes. ^*^P < 0.05; ^**^P < 0.01

To clarify the cellular mechanisms underlying pFSH-induced follicular development, small antral follicles (diameter, 4 ± 0.2 mm) were mechanically isolated from ovaries and subjected to 10 × single-cell sequencing (Fig. 2a). After quality control, 49,638 cells were retained (Table S2). Following principal component analysis, cells were projected onto two dimensions using UMAP, yielding 29 transcriptionally distinct clusters (Fig. 2a). Clusters were annotated to known ovarian cell types on the basis of canonical marker gene expression (Table S3). In total, nine cell types were identified, including: granulosa cells, theca cells and stroma cells, smooth muscle cells, endothelial cells, steroidogenic cells, epithelial cells, T lymphocytes, macrophage/monocyte cells and neuronal cells (Fig. 2b, Table S4). Expression of representative lineage-defining marker genes across the nine cell types is shown in Fig. 2c. As expected, FSHR is exclusively expressed in granulosa cells, which is consistent with existing findings (Fig. 2d).

Cell proportion analysis revealed significant changes in the abundance of five cell types following pFSH treatment: GCs, theca/stroma cells, endothelial cells, T lymphocytes, and macrophage/monocyte cells (Fig. 2e). Among them, the proportion of granulosa cells was significantly increased following pFSH treatment (*P* = 0.0013), rising from 7.26% in the control group to 12.53%. Differential expression analysis was next performed separately for each of these five cell types. A total of 3,729, 3,688, 636, 1,527, and 5,234 differentially expressed genes (DEGs) were identified in theca/stroma cells, endothelial cells, T lymphocytes, macrophage/monocyte cells, and GCs, respectively (Fig. 2f). Notably, GCs displayed the most extensive transcriptional remodeling in response to pFSH treatment.

**Fig. 3:**
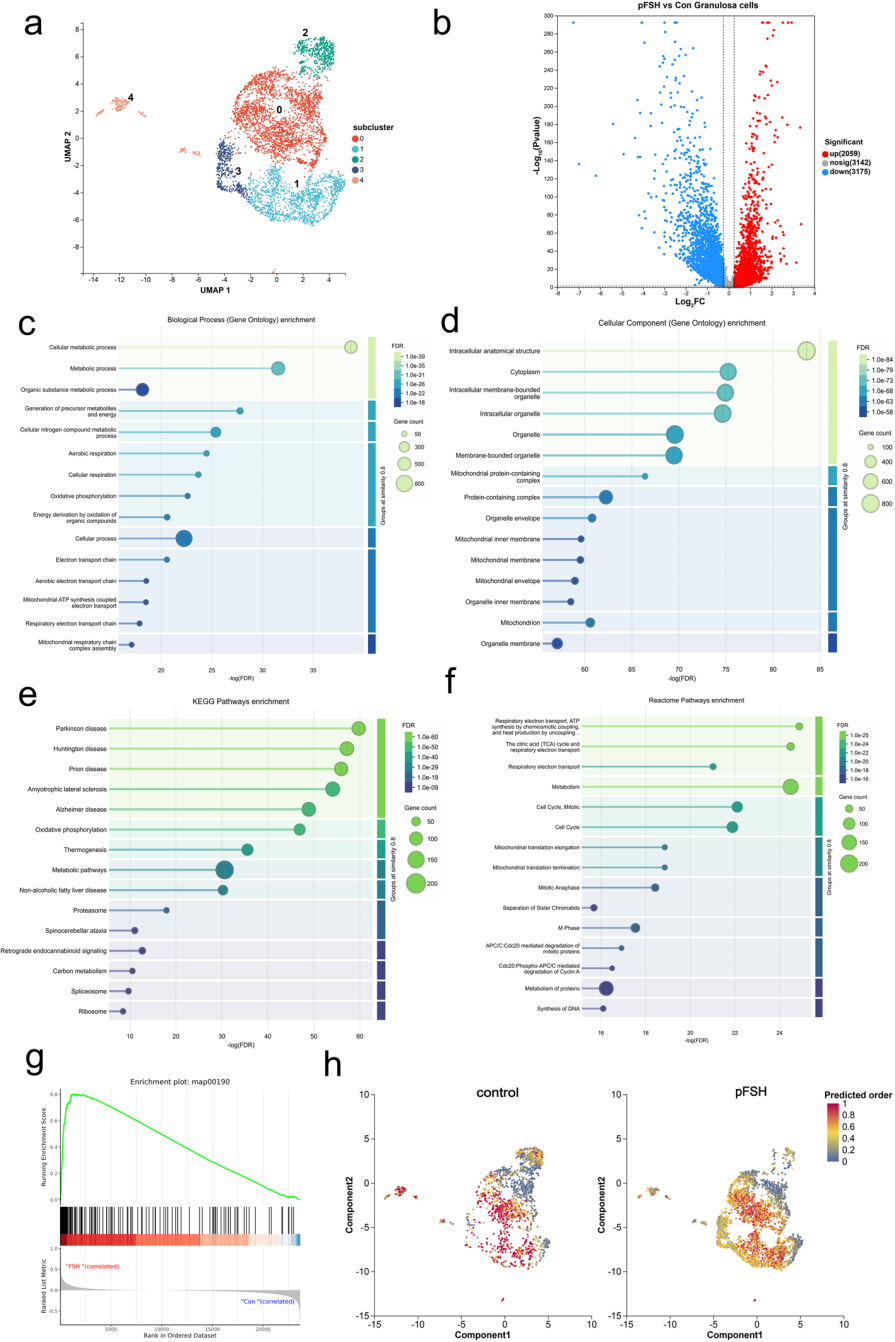
GC transcriptome analysis. **a** UMAP scatterplot visualizing granulosa cell subsets. Each point corresponding to a single cell is color-coded according to its cell type membership. **b** Volcano plot of significant DEGs in GCs from the pFSH-treated group as compared to controls. **c** and **d** GO enrichment analysis of significant DEGs in GCs. **e** KEGG pathway enrichment analysis of significant DEGs in GCs. **f** Reactome pathway enrichment analysis of significant DEGs in GCs. **g** Gene Set Enrichment Analysis of significant DEGs in GCs. **h** UMAP scatter plot illustrating the differentiation potential of granulosa cells. CytoTRACE scores were ranked and scaled between 0 and 1. The score represents the predicted order of the relative differentiation state of cells (0: more differentiated; 1: less differentiated). Cell types with lower differentiation degrees are considered the origin cells

GCs, the primary targets of FSH signaling, were further subclustered into five transcriptionally distinct subpopulations (subcluster 0–4) (Fig. 3a), indicating substantial cellular heterogeneity of the small antral follicles. However, due to the limited availability of well-defined molecular markers for subtypes of bovine GCs, precise functional annotation of these subclusters remains challenging. In addition, the GCs used for downstream in vitro experiments were composed of mixed populations derived from follicles, which currently cannot be reliably separated into stable subtype-specific culture systems. Notably, FSH receptor expression was broadly detected across all GC subclusters, further supporting a global regulatory effect of FSH on GCs [23]. Given these technical and biological constraints, subsequent analyses were focused on GCs as an integrated functional population to capture the overall response of FSH-target cells during follicular development.

Differential expression analysis revealed extensive transcriptional remodeling in GCs following pFSH treatment (Fig. 3b, Table S5). Strikingly, Gene Ontology, KEGG pathway enrichment, and Reactome analyses all highlighted oxidative phosphorylation and mitochondrial electron transport as the top enriched pathways (Fig. 3c–f). To validate these findings, Gene Set Enrichment Analysis of the DEGs was performed, which revealed significant enrichment of the oxidative phosphorylation and glycolysis pathways, with a trend toward higher expression in the pFSH-treated group (Fig. 3g). These results collectively indicate that pFSH significantly upregulates the transcriptional activity of energy metabolism-related genes in GCs. CytoTRACE analysis further revealed an enrichment of cells with low differentiation potential (high predicted order score) in the pFSH-treated group (Fig. 3h). Together, these results indicate that FSH promotes progression of granulosa cells toward a mature differentiation state.

**Fig. 4:**
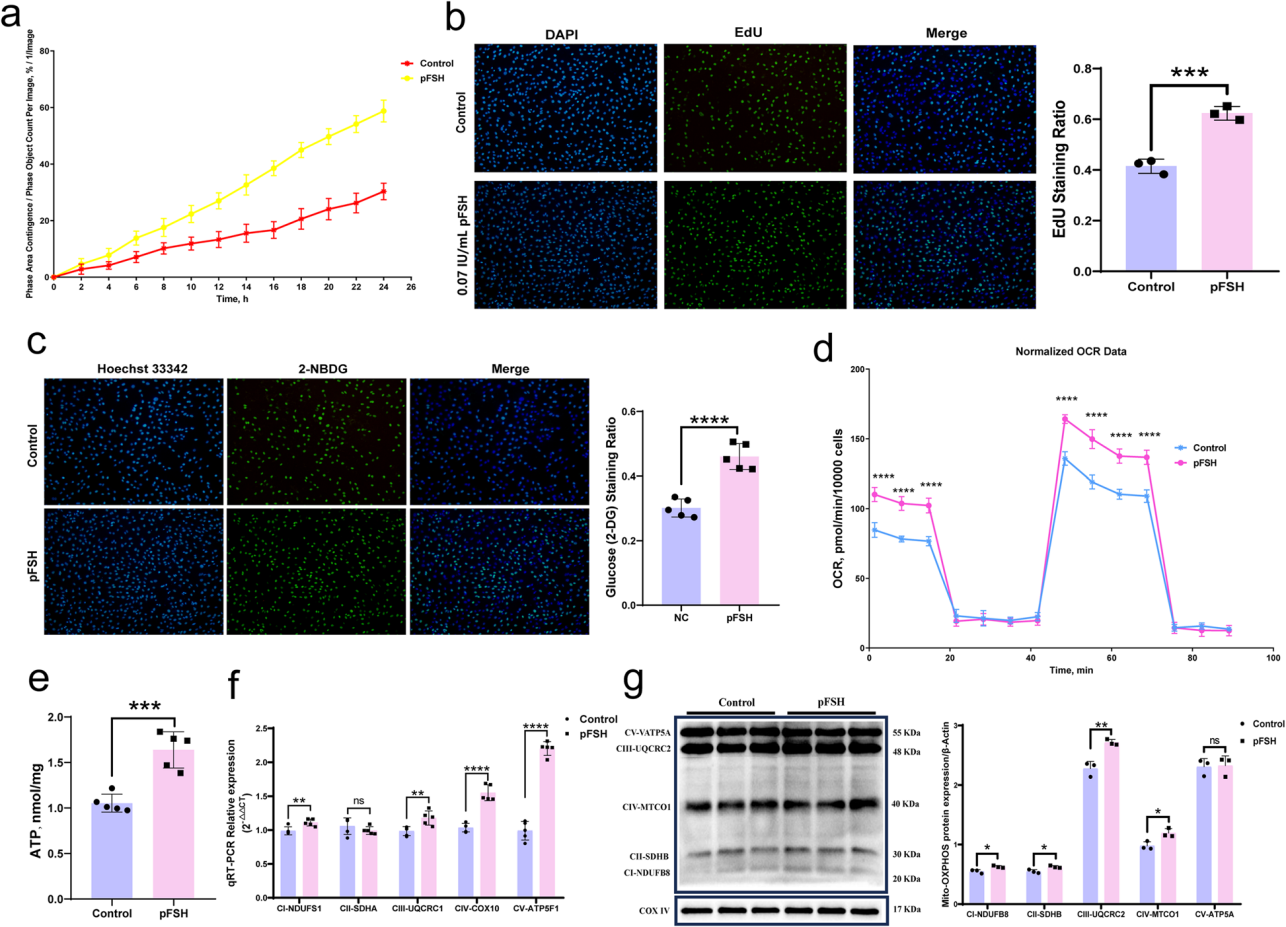
Treatment with pFSH significantly upregulated the proliferation and metabolic activity of GCs. **a** The Incucyte assay showed that treatment with pFSH at 0.07 IU/mL significantly increased the proliferation of GCs (*n* = 5). **b** EdU incorporation assay showing that treatment with pFSH at 0.07 IU/mL significantly increased proliferation of GCs (*n* = 5). **c** Uptake of 2-NBDG was significantly increased in pFSH-treated cells (*n* = 5). **d** ATP levels were significantly increased in pFSH-treated cells (*n* = 5). **e** OCR of control and pFSH-treated GCs under basal conditions and in response to oligomycin, FCCP and antimycin A/Rotenone, showing significantly increased oxidative phosphorylation at 12 h after pFSH treatment (*n* = 5). **f** Expression levels of respiratory chain complex-related genes in GCs treated with pFSH for 12 h. **g** Expression levels of respiratory chain complex proteins in GCs treated with pFSH for 12 h. ^*^P < 0.05; ^**^P< 0.01; ^***^P < 0.001; ^****^P < 0.0001; ns, non-significant

Subsequently, the effects of pFSH treatment on GC proliferation and energy metabolism were validated at the cellular level. Treatment of GCs with pFSH at 0.07 IU/mL for 12 h resulted in significantly upregulated proliferation, as demonstrated by live-cell imaging and the EdU incorporation assay (Fig. 4a and b). Concurrently, cellular glucose uptake (Fig. 4c) and ATP levels (Fig. 4d) were significantly increased. The Seahorse assay revealed that pFSH treatment significantly increased oxidative phosphorylation in GCs, as evidenced by elevated basal OCR, ATP-linked respiration, and maximal OCR (Fig. 4e). Furthermore, RT-qPCR and Western blot analyses showed that pFSH treatment significantly increased the expression levels of four respiratory chain proteins (Fig. 4f and g). These results indicate that pFSH promotes the proliferation and energy metabolism of GCs.

### FOXO1 functions as a negative regulator of GC proliferation and metabolic activity

**Fig. 5:**
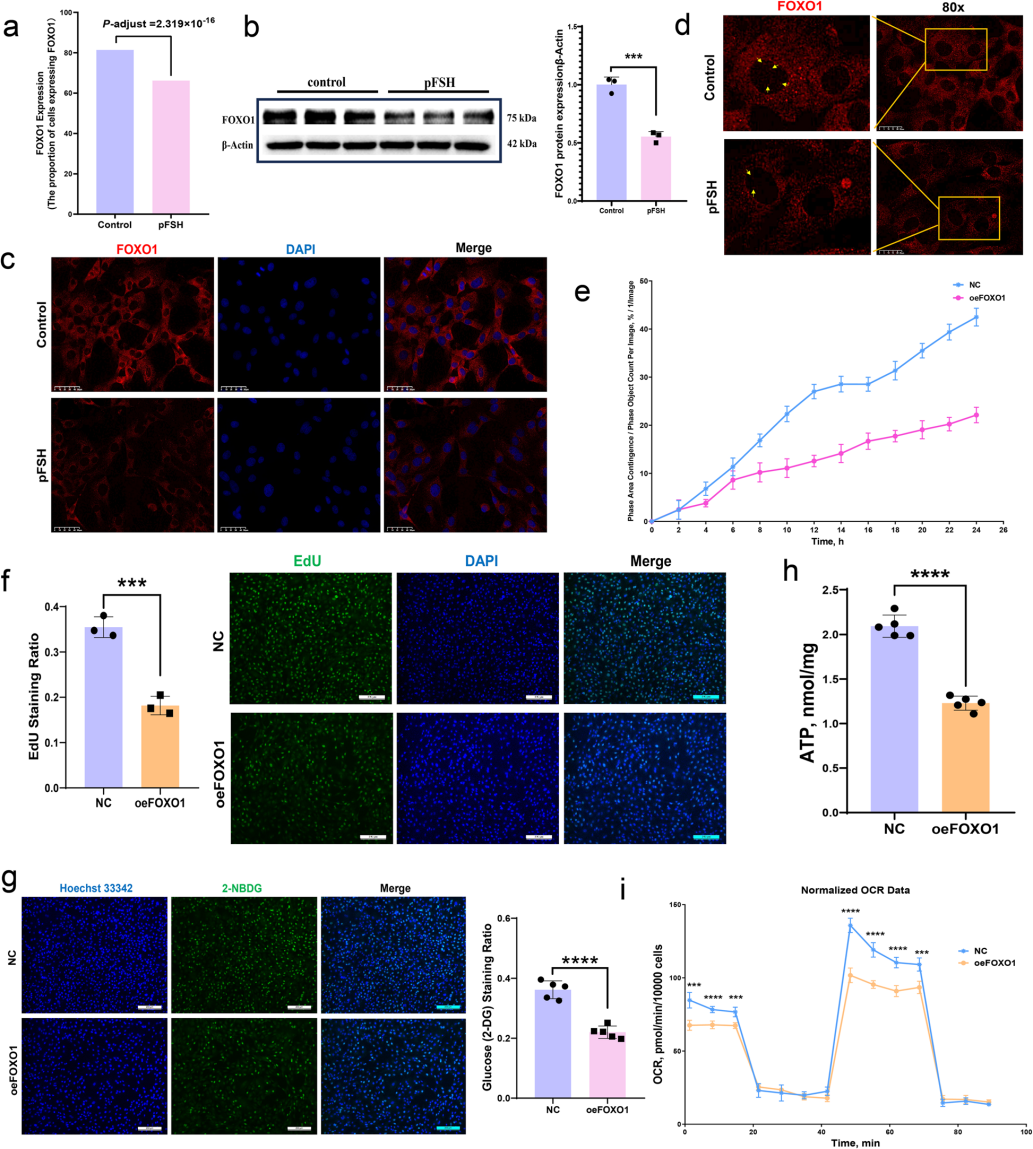
FOXO1 overexpression inhibited GC proliferation and energy metabolism. **a** Bar graph showing FOXO1 expression in the control and pFSH-treated groups as determined by transcriptome sequencing. **b** Western blot analysis (*n* = 3). **c** Immunofluorescence staining (40 ×). **d** Nuclear-cytoplasmic distribution of FOXO1 in GCs at 12 h after pFSH treatment (80 ×). **e** Incucyte assay (*n* = 5). **f** EdU incorporation assay (*n* = 5). **g** Uptake of 2-NBDG was significantly decreased in the oeFOXO1 group (*n* = 5). **h** ATP levels were significantly decreased in the oeFOXO1 group (*n* = 5). **i** OCR of the control and oeFOXO1 groups under basal conditions and in response to FCCP and antimycin A/Rotenone, showing significantly decreased oxidative phosphorylation in the oeFOXO1 group (*n* = 5). ^***^P < 0.001; ^****^P < 0.0001

To investigate the molecular mechanism employed by pFSH to regulate GC proliferation and energy metabolism, an in-depth analysis of the single-cell sequencing dataset was performed, which identified FOXO1 as a candidate transcription factor. Treatment with pFSH significantly reduced FOXO1 expression (Fig. 5a). Furthermore, existing studies indicate that FOXO1 is a key regulator of energy metabolism in various cell types and is involved in regulating cell proliferation [15, 24]. Therefore, we hypothesized that pFSH regulates GC proliferation and energy metabolism through FOXO1. To test this hypothesis, we first examined whether pFSH influences FOXO1 levels in GCs. Western blot and immunofluorescence analyses showed that FOXO1 protein levels were significantly downregulated at 12 h after pFSH treatment (Fig. 5b and c). Furthermore, immunofluorescence revealed that nuclear FOXO1 levels were significantly lower in pFSH-treated cells than the controls (Fig. 5d), indicating that pFSH treatment downregulated FOXO1 and may induce nuclear export. To determine whether FOXO1 regulates GC proliferation and energy metabolism, FOXO1 was overexpressed in GCs by plasmid transfection (oeFOXO1). Incucyte live-cell imaging and the EdU assay showed that, as compared to controls, oeFOXO1 cells exhibited significantly decreased proliferation (Fig. 5e and f), glucose uptake (Fig. 5g), and ATP levels (Fig. 5h). The Seahorse assay demonstrated that FOXO1 overexpression significantly reduced oxidative phosphorylation in GCs, accompanied by a decrease in ATP-linked respiration (Fig. 5i). These results indicate that FOXO1 inhibits GC proliferation and energy metabolism. We next investigated whether pFSH could counteract the inhibitory effects of FOXO1 overexpression. Co-treatment with pFSH significantly rescued the decreased proliferation, glucose uptake, ATP levels, and oxidative phosphorylation induced by FOXO1 overexpression (Fig. S3a–e).

### pFSH promoted FOXO1 phosphorylation via the PI3K/AKT signaling pathway

**Fig. 6:**
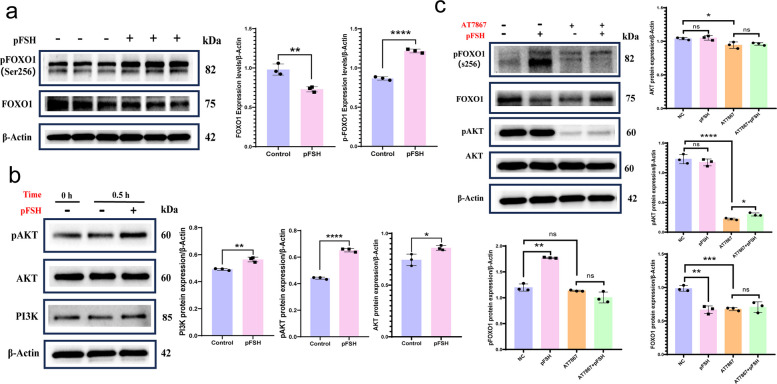
pFSH promoted FOXO1 phosphorylation via the PI3K/AKT signaling pathway. **a** pFOXO1(Ser256) protein levels in GCs treated with pFSH at 0.07 IU/mL for 12 h (*n* = 3). **b** Western blot analysis of PI3K, AKT, and pAKT protein levels in GCs following pFSH treatment (*n* = 3). **c** Protein levels of pFOXO1, FOXO1, pAKT, and AKT in GCs treated with the AKT inhibitor AT7867 and/or pFSH (*n* = 3). ^*^P < 0.05; ^**^P < 0.01; ^***^P < 0.001; ^****^P < 0.0001; ns, non-significant

Surprisingly, pFSH treatment for 12 h partially alleviated the inhibitory effects of FOXO1 overexpression on GC proliferation and energy metabolism (Fig. S3). This finding was not entirely consistent with our initial hypothesis that FOXO1 overexpression would completely block the promoting effects of pFSH on GC proliferation and energy metabolism. Nevertheless, this partial alleviation suggests that FOXO1 is a key regulatory factor of pFSH-mediated modulation of GC function, and that the mode of action may not be solely dependent on downregulation of total protein levels. Given that FOXO1 activity is primarily regulated by phosphorylation, we next examined whether FSH modulates this process. Treatment of GCs with pFSH at 0.07 IU/mL for 12 h significantly increased pFOXO1 (Ser256) levels, as determined by Western blot analysis (Fig. 6a). Given that the PI3K/AKT pathway regulates FOXO1 phosphorylation, PI3K, AKT, and pAKT levels were measured in GCs after pFSH treatment. As compared to controls, the pAKT/AKT ratio and PI3K protein levels were significantly increased after pFSH treatment for 0.5 h (Fig. 6b). Furthermore, treatment with the AKT inhibitor AT7867 markedly decreased the pFOXO1(Ser256)/FOXO1 ratio as compared to pFSH treatment alone. These results indicate that pFSH promoted FOXO1 phosphorylation via the PI3K/AKT pathway in bovine GCs (Fig. 6c).

### FOXO1 phosphorylation is required for pFSH-induced GC proliferation and metabolic activation

**Fig. 7:**
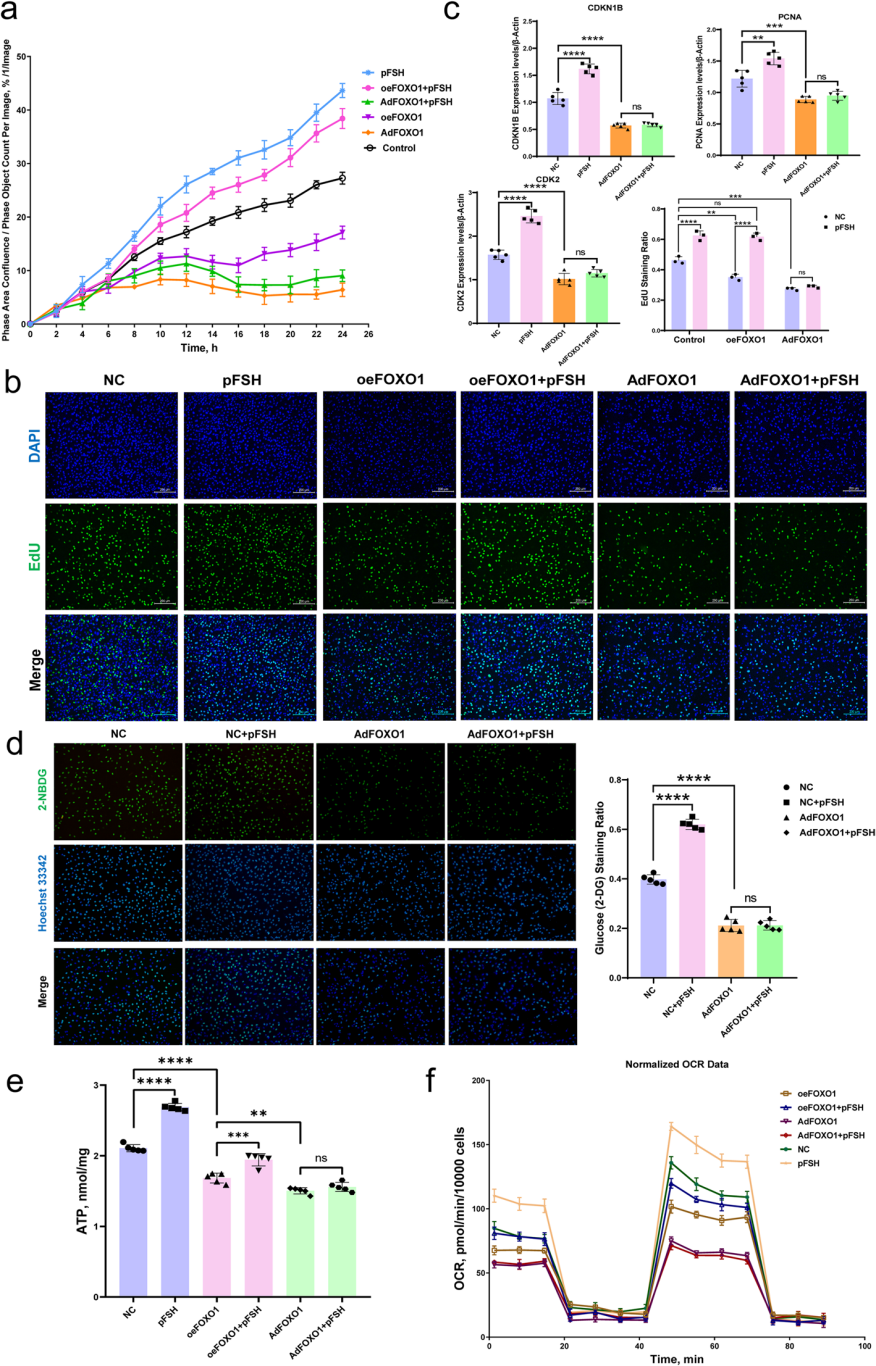
pFSH regulates GC proliferation and metabolism by promoting FOXO1 phosphorylation. **a** Incucyte assay (*n* = 5). **b** EdU incorporation assay (*n* = 5). **c** RT-qPCR analysis of proliferation-associated gene expression (*n* = 5). **d** Uptake of 2-NBDG was significantly decreased in the FOXO1(S267A) group (*n* = 5). **e** ATP levels were significantly decreased in the FOXO1(S267A) group (*n* = 5). **f** OCR was significantly decreased in the FOXO1(S267A) group (*n* = 5). ^**^P < 0.01; ^***^P < 0.001; ^****^P < 0.0001; ns, non-significant

To directly assess the functional importance of FOXO1 phosphorylation, we first constructed the dephosphorylated overexpression vector FOXO1(S267A) with a mutation at the S267 site (corresponding to human Ser256). Incucyte live-cell imaging and the EdU assay showed that overexpression of FOXO1(S267A) significantly inhibited GC proliferation as compared to the negative control (NC), with an even more pronounced effect than oeFOXO1, whereas no significant difference was observed as compared to FOXO1(S267A) + pFSH (Fig. 7a and b). The RT-qPCR results showed significant downregulation of the proliferation-associated genes *CDKN1B, PCNA,* and *CDK2* in the FOXO1(S267A) group, while pFSH treatment following FOXO1(S267A) overexpression did not significantly alter expression levels as compared to the overexpression alone group (Fig. 7c). These results indicate that overexpression of FOXO1(S267A) significantly inhibited cell proliferation and abrogated the pro-proliferative effect of pFSH on GCs. Further assessment of energy metabolism revealed that, as compared to the NC group, overexpression of FOXO1(S267A) significantly decreased glucose uptake (Fig. 7d), ATP levels (Fig. 7e), ATP-linked respiration, and mitochondrial oxidative phosphorylation, with a more pronounced decrease than with oeFOXO1 cells (Fig. 7f). Similarly, no significant differences were observed in these parameters as compared to the FOXO1(S267A) + pFSH group (Fig. 7d–f), indicating that overexpression of FOXO1(S267A) inhibited energy metabolism and abrogated the pro-metabolic effect of pFSH on GCs. These results demonstrate that phosphorylation of FOXO1 at S267 is a critical event required for pFSH to promote GC proliferation and energy metabolism.

### FOXO1 regulated GC proliferation and energy metabolism by promoting ISG15 expression

**Fig. 8:**
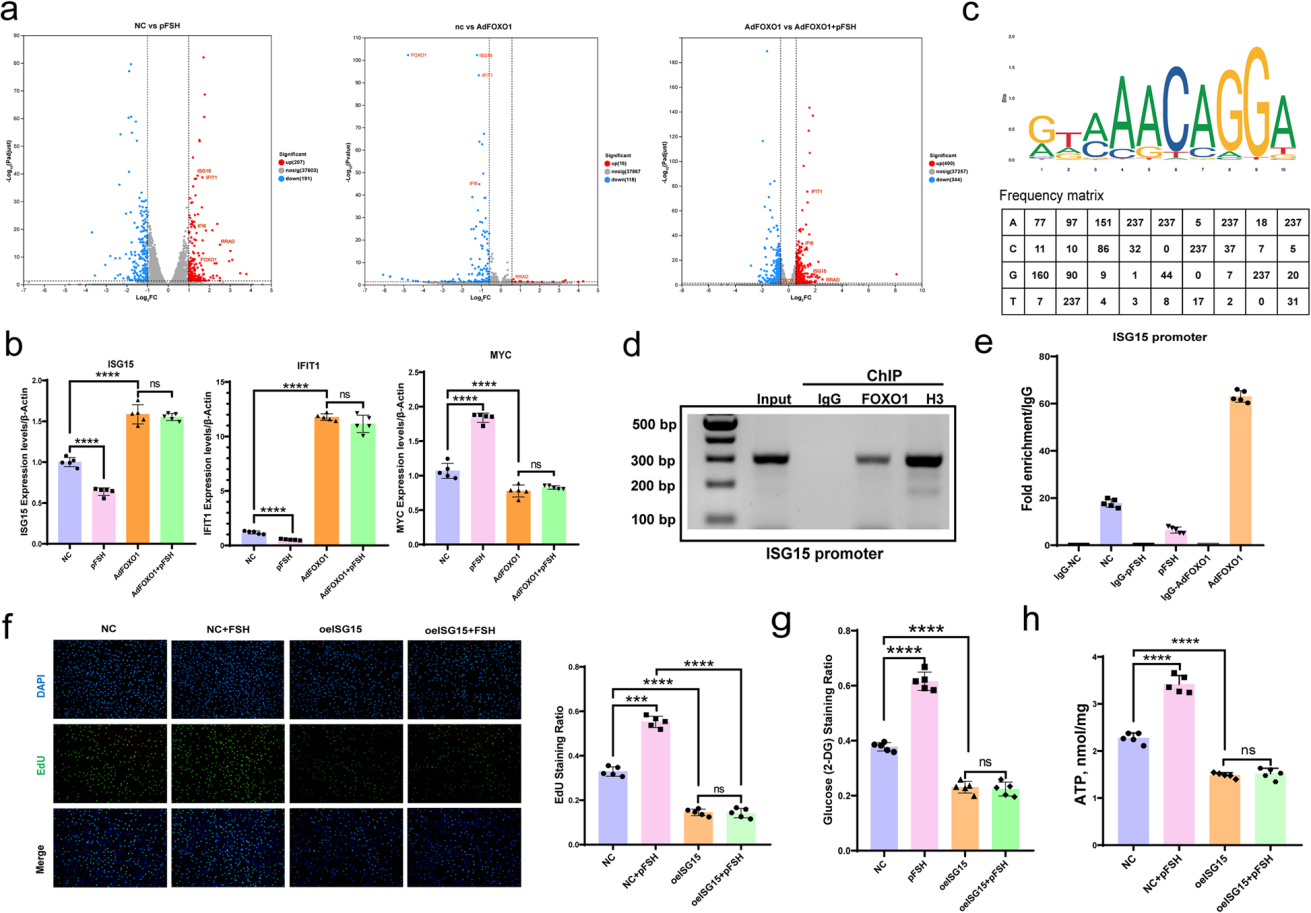
FOXO1 regulated GC proliferation and energy metabolism by promoting ISG15 expression. **a** Volcano plot of significant DEGs. **b** RT-qPCR analysis of ISG15, IFIT1, and MYC gene expression (*n* = 5). **c** JASPAR database analysis predicted FOXO1 binding sites in the ISG15 promoter. **d** ChIP-qPCR analysis showed FOXO1 binding to the ISG15 promoter region. **e** ChIP-RT-qPCR analysis of FOXO1 binding to the identified site in the NC, pFSH-treated, and FOXO1(S267A)-overexpressing groups (*n* = 3). **f** EdU assay assessing cell proliferation (*n* = 5). **g** Uptake of 2-NBDG (*n* = 5). **h** ATP levels (*n* = 5). ^***^P < 0.001; ^****^P < 0.0001; ns, non-significant

To identify the downstream targets of FOXO1, we performed transcriptome sequencing analysis of four groups of GCs: an NC group transfected with an empty vector, an empty vector transfection group treated with pFSH, a group transfected with the FOXO1(S267A) mutant, and a group transfected with FOXO1(S267A) and treated with pFSH. Comparisons of the NC vs. FOXO1(S267A), NC vs. pFSH, and FOXO1(S267A) vs. FOXO1(S267A) + pFSH groups identified 134, 398, and 191 significant DEGs, respectively. Further analysis revealed that ISG15 was consistently among the top 15 DEGs in each comparison group. Notably, ISG15 was significantly upregulated in the FOXO1(S267A) group as compared to the NC group, and significantly downregulated by pFSH treatment in both the NC vs. pFSH and FOXO1(S267A) vs. FOXO1(S267A) + pFSH comparisons (Fig. 8a), suggesting that ISG15 might be a key target gene downstream of FOXO1 regulation. RT-qPCR validation using randomly selected genes (ISG15, IFIT1, MYC, and RRAD) confirmed the transcriptome results, indicating the reliability of the sequencing data (Fig. 8b).

JASPAR database analysis predicted potential FOXO1 binding sites in the ISG15 promoter region (Fig. 8c). Chromatin immunoprecipitation (ChIP)-RT-qPCR confirmed that FOXO1 binds to the ISG15 promoter region between 51,466,177 and 51,466,184 (Fig. 8d, Fig. S3b). We designed primers targeting the FOXO1 binding site in the ISG15 promoter and performed ChIP-RT-qPCR to measure FOXO1 occupancy in NC, pFSH-treated, and FOXO1(S267A)-overexpressing cells. As compared to the NC group, FOXO1 binding was significantly reduced by pFSH treatment, but markedly increased in FOXO1(S267A)-overexpressing cells relative to both other groups (Fig. 8e). This indicates that pFSH, via regulation of FOXO1 phosphorylation levels, affects binding capacity to the ISG15 promoter, thereby regulating ISG15 expression levels. To clarify the role of ISG15 in GC proliferation and energy metabolism, and to determine whether this gene is a key target in the pFSH-regulated pathway of cell proliferation and energy metabolism, ISG15 was overexpressed in GCs via plasmid transfection. The EdU assay showed that, as compared to controls, ISG15-overexpressing cells exhibited significantly decreased proliferation (Fig. 8f), glucose uptake (Fig. 8g), and ATP levels (Fig. 8h), while pFSH treatment did not rescue these phenotypes (Fig. 8f–h). In brief, pFSH promoted GC proliferation and energy metabolism by inducing FOXO1 phosphorylation, thereby relieving FOXO1-mediated transcriptional activation of ISG15.

## Discussion

Follicular development is a highly coordinated process that integrates endocrine, paracrine, and metabolic signals. Although FSH, as a key hormone driving follicular growth, has been widely applied in controlled ovarian stimulation protocols for humans and superovulation protocols for livestock, the substantial inter-individual variability in response to exogenous FSH remains a long-standing challenge. The core issue lies in the unclear molecular mechanism by which FSH promotes synchronous multi-follicular development. To elucidate this mechanism, the present study established an in vivo model of pFSH-induced multi-follicular development using Holstein cows. Combined with in vitro functional validation experiments in GCs, we identified a previously unrecognized FSH–FOXO1–ISG15 signaling axis that links hormonal stimulation to metabolic reprogramming in GCs, thereby providing a mechanistic framework for FSH-driven multi-follicular development.

Using a bovine model of pFSH-induced multi-follicular development, we demonstrate that exogenous pFSH overrides the physiological “FSH threshold/window” constraint, leading to activation of multiple follicles. The observed increases in ovarian weight, antral follicle number, and circulating estradiol levels collectively indicate enhanced GC activity and follicular growth. Estradiol is primarily synthesized by GCs in antral follicles, and its synthesis and release are closely associated with the progression of follicular development and the number of GCs [25]. The increased estradiol levels observed in this study indicate that under pFSH stimulation, more follicles are in a developmental state and GC function is activated, which is consistent with the morphological differences observed in ovarian weight and follicle numbers. Notably, the elevation in progesterone levels is consistent with previous observations in super-ovulated cattle and may reflect partial luteinization under sustained FSH stimulation [26]. Furthermore, given that progesterone is an important precursor for estradiol synthesis, the moderate increase in progesterone levels following pFSH treatment is consistent with the enhanced estradiol production by GCs. These findings validate the physiological relevance of our model, thereby providing a robust platform for mechanistic investigations.

A key finding of this study is that FSH induced global metabolic reprogramming of GCs. Single-cell transcriptomic analysis revealed that oxidative phosphorylation and mitochondrial electron transport were the most prominently enriched pathways following pFSH treatment. In vitro functional validation experiments further confirmed that pFSH treatment significantly upregulated GC proliferation, as well as enhanced cellular glucose uptake, oxidative phosphorylation, ATP synthesis, and other processes related to energy metabolism, consistent with the single-cell transcriptome findings. As the core carbon source for both biosynthetic and energy metabolism, increased glucose uptake provides more substrate for mitochondrial oxidative phosphorylation to generate ATP [27, 28]. Oxidative phosphorylation, being the primary pathway for cellular ATP production, ensures an adequate energy supply for the cell [29]. Beyond providing energy for cell proliferation, metabolic intermediates also serve as building blocks for nucleotides, amino acids, and other macromolecules [30, 31]. Given that GCs provide essential metabolic support to the oocyte, these findings suggest that FSH-driven metabolic activation not only fuels GC proliferation but also establishes a metabolically permissive microenvironment for follicular development. Thus, metabolic reprogramming emerged as a central mechanism underlying synchronous multi-follicular growth.

At the molecular level, FOXO1 was identified as a critical regulatory node linking FSH signaling to metabolic control. FOXO1 is a well-established transcription factor involved in regulating cell proliferation, metabolism, and apoptosis [32], and its transcriptional activity is tightly controlled by post-translational modifications, particularly phosphorylation [33, 34]. In the dephosphorylated state, FOXO1 localizes to the nucleus and activates downstream target genes. Upon activation of kinase pathways, such as PI3K/AKT or MAPK/ERK, FOXO1 is phosphorylated at key sites (Thr24, Ser256, and Ser319 in mammals). Phosphorylated FOXO1 then binds to 14-3-3 proteins, translocating from the nucleus to the cytoplasm, where it is subsequently degraded via the ubiquitin–proteasome pathway, thereby losing transcriptional activity [21]. In recent years, the regulatory role of FOXO1 in the reproductive system, particularly in ovarian follicular development, has garnered significant attention [35–37]. We showed that pFSH activates the PI3K/AKT pathway to induce FOXO1 phosphorylation at Ser267, leading to nuclear exclusion and functional inactivation. This finding is consistent with a prior study of ovine GCs [32]. Notably, overexpression of the dephosphorylated FOXO1(S267A) mutant significantly abrogated the promoting effects of pFSH on bovine GC proliferation and energy metabolism, with an even more pronounced inhibitory effect as compared to overexpression of wild-type FOXO1, indicating that FOXO1 phosphorylation at Ser267 is a critical molecular event required for pFSH function in bovine GCs. Following nuclear export, phosphorylated FOXO1 is likely degraded via the ubiquitin–proteasome pathway, which explains the significant downregulation of total FOXO1 protein levels observed after pFSH treatment. These findings extend previous observations in other species and establish FOXO1 as a central mediator of FSH-induced GC activation. Beyond the PI3K/AKT–FOXO1 pathway identified in this study, FSH has been shown to regulate granulosa cell function via several other intracellular signaling cascades. These include the cAMP/PKA pathway, which is central to steroidogenesis and follicular development [38]; the MAPK/ERK pathway, which stimulates cell proliferation, differentiation, and survival [39], and the mTOR signaling pathway, regulating cellular metabolism and follicular growth [40]. Future studies could further explore the potential interactions among these signaling pathways and their underlying regulatory mechanisms.

Our study further uncovered ISG15 as a direct downstream target of FOXO1, revealing an unexpected link between endocrine signaling and interferon-related pathways. ISG15, functioning as a ubiquitin-like modifier, is widely involved in cellular processes, including immune regulation, proliferation, and energy metabolism [41–44]. Existing studies have shown that in human hepatic stellate cells, downregulation of ISG15 activates the TGFβ2/SMAD2 pathway, thereby promoting cell proliferation and activation [45]. Consistently, Chen et al. further demonstrated that silencing ISG15 in mouse GCs significantly upregulated energy metabolism and follicular responsiveness to gonadotropins [46]. Recent research indicates that ISG15 is aberrantly highly expressed in GCs of COVID-19 patients, leading to metabolic disorders and subsequent decline in oocyte quality [47]. These reports suggest and FOXO1 may influence GC proliferation and energy metabolism by downregulating ISG15. The results of the present study demonstrate that FOXO1 directly binds to the ISG15 promoter and promotes transcription, whereas FSH-induced FOXO1 phosphorylation suppresses this interaction, leading to reduced ISG15 expression. Considering that loss of function for ISG15 promotes granulosa cell proliferation, and that FSH is a well-established promoter of proliferation, thus overexpression of ISG15 was used as the primary functional approach to determine whether ISG15 mediates FOXO1-dependent regulation of granulosa cell energy metabolism and proliferation under pFSH treatment. Functional validation experiments demonstrated that ISG15 overexpression significantly inhibited GC proliferation, glucose uptake, and ATP synthesis. Furthermore, pFSH treatment failed to rescue these inhibitory effects in ISG15-overexpressing cells, suggesting that ISG15 acts as a critical downstream effector of the pFSH–FOXO1 signaling axis.

**Fig. 9:**
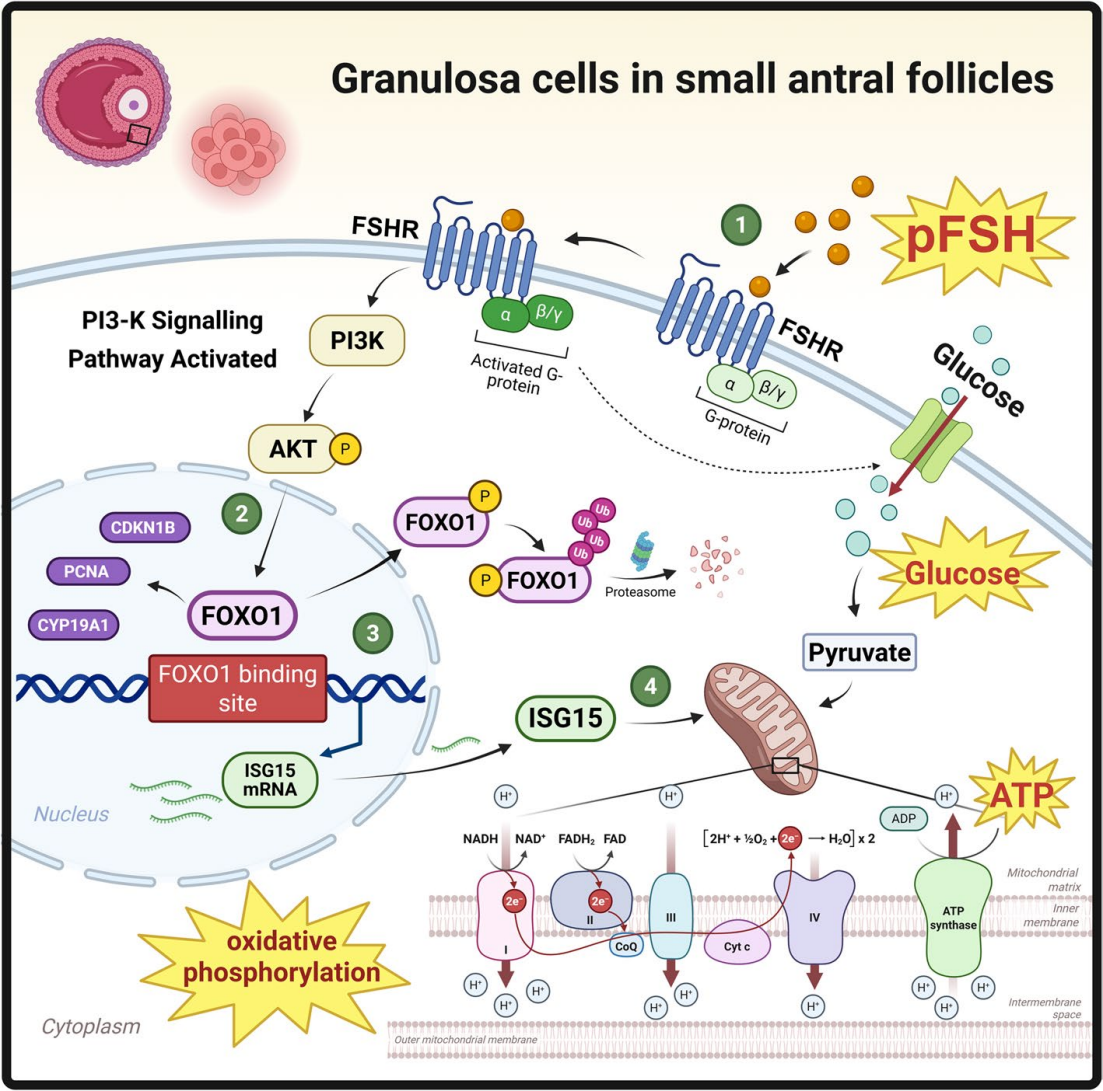
Regulatory role of the pFSH–FOXO1–ISG15 axis in GC proliferation and energy metabolism

Notably, single-cell analysis revealed transcriptional heterogeneity within GCs, with multiple subpopulations identified. However, due to the lack of well-defined molecular markers for bovine GC subtypes and the current limitations in establishing subtype-specific in vitro models, our study focused on GCs as an integrated functional population. The broad expression of the FSH receptor across all GC subclusters supports a global regulatory role of FSH, suggesting that metabolic reprogramming represents a coordinated response across GC populations. Future studies combining lineage tracing and subtype-specific functional analyses will be required to resolve the contributions of distinct GC subpopulations. In addition to granulosa cells, the most pronounced changes in cell proportions following pFSH treatment were observed in T cells and macrophages, followed by endothelial cells, theca cells, and stromal cells, according to the single-cell RNA-seq analysis. Increasing evidence suggests that follicular development depends on the coordinated regulation of multiple cell populations within the ovarian microenvironment [48]. T cells and macrophages, as key immune cells, regulate local inflammatory responses, tissue remodeling, angiogenesis, and intercellular communication, thereby maintaining ovarian homeostasis and participating in follicular development [49, 50]. In parallel with these immune cells, theca cells and stromal cells contribute to steroid hormone synthesis and extracellular matrix remodeling [5, 51, 52], while endothelial cells play essential roles in follicular angiogenesis, nutrient supply, and hormone transport [53]. The observed changes in the proportions of these cell populations suggest that pFSH may promote follicular development not only through the direct regulation of granulosa cell function, but also by remodeling the local ovarian microenvironment and coordinating multicellular networks involved in follicular development. Further studies are required to investigate the interactions among different cell populations and to clarify their specific roles in pFSH-induced follicular development Fig. 9.

## Conclusions

In summary, this study elucidated the molecular mechanism by which pFSH promotes synchronous multi-follicular development through the FOXO1–ISG15 regulatory axis governing GC proliferation and energy metabolism. This work provides a conceptual framework to clarify how endocrine signals are integrated with metabolic and immune pathways to regulate follicular development. From a translational perspective, targeting metabolic pathways or the FOXO1–ISG15 axis may offer new strategies to improve the efficiency and consistency of assisted reproduction and livestock superovulation protocols.

## Supplementary Information

Additional file 1: Table S1. Primer sequences for RT-qPCR. Table S2. Results of Cell quality filtering and evaluation. Table S3. Differentially expressed genes of each cluster in small antral follicles. Table S4. Signature genes used to annotate cell types. Table S5. Significantly differentially expressed genes in pFSH-treated granulosa cells.

Additional file 2: Fig. S1. Optimization and validation of FOXO1 overexpression efficiency in bovine granulosa cells. Fig. S2 Results of ovarian development monitoring. Fig. S3 pFSH partially alleviated the inhibitory effects of FOXO1 overexpression on GC proliferation and energy metabolism.

Additional file 3: Original images for Western blot.

## Data Availability

The raw Genomics scRNA-seq data generated in this study have been deposited in the NCBI Sequence Read Archive (SRA, https://www.ncbi.nlm.nih.gov/sra). Apart from the datasets included in the article/supplementary materials, all other data generated in this study are available from the corresponding author upon request.

## Abbreviations

CIDR: Controlled Internal Drug Release
ChIP: Chromatin Immunoprecipitation
DEGs: Differentially expressed genes
EdU: 5-Ethynyl-2'-deoxyuridine
FBS: Fetal bovine serum
FCCP: Carbonyl cyanide-p-trifluoromethoxy phenylhydrazone
FOXO1: Forkhead box protein O1
GCs: Granulosa cells
OCR: Oxygen consumption rate
pFSH: Porcine purified pituitary FSH
PS: Penicillin-streptomycin
t-SNE: T-Distributed Stochastic Neighbor Embedding
UMAP: Uniform Manifold Approximation and Projection
2-NBDG: 2-Deoxy-2-{(7-nitro-2,1,3-benzoxadiazol-4-yl)amino}-D-glucose
