# Supplementary material for: Follicle-stimulating hormone promotes energy metabolism in bovine granulosa cells through the FOXO1-ISG15 pathway to maintain the development of multiple follicles
Source: J Anim Sci Biotechnol. 2026 Aug 12;17:164. doi: 10.1186/s40104-026-01486-9 (PMC13465301; doi:10.1186/s40104-026-01486-9)
Supplement: Supplementary file 1 [file 40104_2026_1486_MOESM2_ESM.docx]

**Additional file 2:**

**Fig. S1** Results of ovarian development monitoring. The figure shows ultrasound images taken one day before the experimental treatment and two days after pFSH injection, as well as the ovarian morphology on the day of slaughter

**Fig. S2** Optimization and validation of FOXO1 overexpression efficiency in bovine granulosa cells. **a** Representative bright-field images showing the morphology and viability of GCs 12 h after transfection with empty vector (NC) or different doses of the FOXO1 overexpression plasmid (0.5, 1.0, and 2.5 μg). **b** Relative FOXO1 mRNA expression levels in GCs 12 h after transfection with different doses of the FOXO1 overexpression plasmid, as determined by RT-qPCR (*n =* 3 independent experiments). **c** FOXO1 protein expression levels in GCs 12 h after transfection with different doses of the FOXO1 overexpression plasmid, as determined by western blot analysis (*n =* 3 independent experiments). Based on the transfection efficiency, expression level, and cell morphology, 1.0 μg of FOXO1 overexpression plasmid was selected for all subsequent overexpression experiments. Statistical analysis was performed by Two-tailed Student’s *t*-test. ^*^*P* < 0.05; ^**^*P* < 0.01; ^***^*P* < 0.001; ^****^*P* < 0.0001; ns: non-significant

**Fig. S3** pFSH partially alleviated the inhibitory effects of FOXO1 overexpression on GC proliferation and energy metabolism. **a** and **b** Treatment with pFSH significantly alleviated the inhibitory effect of FOXO1 overexpression on cell proliferation. **a** Incucyte assay (*n =* 5). **b** EdU incorporation assay (*n =* 5). **c–e** Treatment with pFSH significantly alleviated the inhibitory effect of FOXO1 overexpression on cellular energy metabolism. **c** Uptake of 2-NBDG (*n =* 5). **d** ATP levels (*n =* 5). **e** Oxidative phosphorylation as determined with the Seahorse assay (*n =* 5)
