# Supplementary material for: Follicle-stimulating hormone promotes energy metabolism in bovine granulosa cells through the FOXO1-ISG15 pathway to maintain the development of multiple follicles
Source: J Anim Sci Biotechnol. 2026 Aug 12;17:164. doi: 10.1186/s40104-026-01486-9 (PMC13465301; doi:10.1186/s40104-026-01486-9)
Supplement: Supplementary file 2 [file 40104_2026_1486_MOESM3_ESM.docx]

Additional file 3:

Figure 4g Expression levels of respiratory chain complex proteins in GCs treated with pFSH for 12 h

(1) respiratory chain complex proteins

(2) cox IV

Figure 5b FOXO1 protein levels were significantly downregulated in GCs at 12 h after pFSH treatment (n = 3).

(1) pFOXO1 (Ser256)

(2) β-Actin

Figure 6a pFOXO1 (Ser256) protein levels in GCs treated with pFSH at 0.07 IU/mL for 12 h (n = 3)

(3) pFOXO1 (Ser256)

(4) FOXO1
(5) β-Actin

Figure 6b Western blot analysis of PI3K, AKT, and pAKT protein levels in GCs following pFSH treatment (n = 3)

(1) pAKT

(2) AKT

(3) PI3K

(4) β-Actin

Figure 6c Protein levels of pFOXO1, FOXO1, pAKT, and AKT in GCs treated with the AKT inhibitor AT7867 and/or pFSH (n = 3)

(1) pFOXO1 (Ser256)

(2) FOXO1

(3) pAKT

(4) AKT
(5) β-Actin

Figure 8d ChIP-PCR analysis showed FOXO1 binding to the *ISG15* promoter region (This figure shows the CHIP-PCR validation results of multiple candidate genes. The red box highlights the detection result of *ISG15*, while the others are the detection results of *IFIT1, IFI6,* and *MYC* respectively.
